# Inhibition of *Candida albicans* in vivo and in vitro by antimicrobial peptides chromogranin A-N12 through microRNA-155/suppressor of cytokine signaling 1 axis

**DOI:** 10.1080/21655979.2021.2017680

**Published:** 2022-01-16

**Authors:** Xiaohua Li, Qun Hu, Qiong Lin, Jianxiong Luo, Junping Xu, Lifang Chen, Liyu Xu, Xin Lin

**Affiliations:** a Department of Pulmonary and Critical Care Medicine, Affiliated Fuzhou First Hospital of Fujian Medical University, Fuzhou, Fujian, China; b Department of Pulmonary and Critical Care Medicine, 900 Hospital of the Joint Logistics Team, Fuzhou, Fujian, China; c Department of Respiratory, Critical Care, and Sleep Medicine Xiang’an Hospital of Xia Men University, Xiamen, China

**Keywords:** *Candida albicans*, CGA-N12, SOCS1, antimicrobial peptides, miR-155

## Abstract

Antimicrobial peptides (AMPs) have proven to inhibit a variety of pathogens. Chromogranin A-N12 (CGA-N12) is a kind of AMP, and it is characterized by stable structure, high anti-Candida activity, and good safety. However, it remains unclear whether CGA-N12 could effectively inhibit the growth of *Candida albicans (C. albicans)*. Colony forming assays were used to measure minimal inhibitory concentration (MIC), minimal fungicidal concentration (MFC), and time-kill curve. Disseminated *C. albicans* rabbit model was established to investigate the influence of CGA-N12 on histological damage. The protein and mRNA levels of suppressor of cytokine signaling 1 (SOCS1) after treatment were investigated. The MIC and MFC of CGA-N12 against *C. albicans* was 6 mg/mL. CGA-N12 considerably inhibited germ tube formation of *C. albicans*. The fungal load in the tissues and inflammatory factors in the serum were suppressed by CGA-N12. CGA-N12 significantly reduced the histological changes caused by *C. albicans*, and the protein and mRNA levels of SOCS1 were markedly inhibited. The inhibition effect of CGA-N12 on *C. albicans* and significant improvement of histological damage by CGA-N12 through microRNA-155/SOCS1 axis were proved in this study. This study proposes a novel therapeutic strategy for the treatment and prevention of *C. albicans*.

**Abbreviations:** AMPs: Antimicrobial peptides; MIC: Minimal inhibitory concentration; MFC: Minimal fungicidal concentration; AIDS: Acquired immune deficiency syndrome; PBS: Phosphate buffer saline; FBS: Fetal bovine serum; ROS: Reactive oxygen species; CFU: Colony formation unit; CGA: Chromogranin A; SOCS1: Suppressor of cytokine signaling 1; SDA: Sabouraud Dextrose Agar; GRAVY: Grand average of hydropathicity; *C. parapsilosis: Candida parapsilosis; C. albicans: Candida albicans*

## 1. Introduction

*Candida* is a common fungus, which mainly exists in oropharynx, gastrointestinal tract and vaginal mucosal surface [1]. It could invade the mucosal epithelium and vascular endothelium, causing hematogenous disseminated infection in susceptible people, leading to opportunistic fungal infection [2]. With the development of organ transplantation technology, the increase of acquired immune deficiency syndrome (AIDS) incidence rate and the use of broad-spectrum antibiotics, *Candida albicans (C. albicans)* became one of the most common pathogens causing iatrogenic infection in immunodeficiency populations, and the incidence and mortality rates are increasing significantly in the world. Epidemiological investigation indicated that Candida is the fourth most common pathogen of iatrogenic sepsis, with a mortality rate of nearly 50% [3,4]. Amphotericin B, itraconazole and fluconazole are commonly used as antifungals, but serious side effects limit their application [5]. Meanwhile, *C. albicans* can easily develop resistance toward antifungal agents [6]. Therefore, the development of new anti-*C. albicans* drugs is necessary.

Antimicrobial peptides (AMPs) also known as peptide antibiotics, are a class of small peptides that widely exist in nature and are used by organisms to protect themselves from exogenous pathogenic microorganisms [7,8]. They are composed of 8–70 amino acids and play an important role in innate immunity [9]. AMPs do not only widely resist a variety of pathogens, including bacteria, fungi, viruses, cancer cells, parasites, but also effectively kill bacteria resistant to a variety of antibiotics [10]. AMPs have small molecular weight, simple structure–activity relationship and significant curative effect [11]. Therefore, they are potential new antifungal drugs with great application value.

Chromogranin A (CGA) is a soluble protein distributed in the chromaffin granules of nerve cells and endocrine cells [12]. CGA and its derived peptides have anti-vasoconstrictive, anti-bacterial and anti-fungal properties [13]. Chromogranin A-N46 (CGA-N46), which is composed of 31–76 amino acids at the N-terminal of CGA, has strong antifungal activity [14]. CGA-N46 can reduce the level of reactive oxygen species (ROS) in cells, destroy the stability of mitochondrial membrane potential, and inhibit the synthesis of deoxyribonucleic acid. No hemolysis was observed when the concentration of CGA-N46 is less than 8 mM, and no cytotoxicity to normal cells was found [15–17]. Chromogranin A-N12 (CGA-N12) is a peptide derived from the structural modification of CGA-N46, and compared with CGA-N46, it has the characteristics of stable structure, high anti-*Candida* activity and good safety [18,19]. Therefore, CGA-N12 has great potential to become a new antifungal drug. However, whether CGA-N12 can effectively inhibit *C. albicans* remains unclear.

In this study, the germicidal activity of CGA-N12 against *C. albicans*, the effect of CGA-N12 on germ tube formation rate, and the histological changes caused by *C. albicans* were investigated. We suspected that CGA-N12 might exert an anti-*C. albicans* activity through miR-155/suppressor of cytokine signaling 1 (SOCS1). We aimed to prove the inhibition of CGA-N12 on *C. albicans* in vivo and in vitro through miR-155/SOCS1 axis. This study might provide a novel thought for the preventing and treating *C. albicans* infections.

## 2. Methods

### 2.1. *Preparation of CGA-N12 and* C. albicans

CGA-N12 was synthesized by the solid-phase peptide synthesis method with C-terminal and N-terminal de-protection as described previously [14,20]. Peptide purification was conducted using high-performance liquid chromatography. The purification yield of CGA-N12 is around 16%. The amino acid sequence of CGA-N12: NH2–ALQGAKERAHQQ–COOH. The physicochemical properties of CGA-N12 is listed as below: molecular weight: 1336.4, PI: 8.8, grand average of hydropathicity (GRAVY): −1.4 (CGA-N12 is hydrophilic), half-life (Mammalian reticulocytes: 4.4 h, yeast: >20 h, E. coli: >10 h), instability index: 38.23, aliphatic index: 57.5 [14,21].

*C. albicans* (ATCC10231) was transferred twice on Sabouraud Dextrose Agar (SDA) medium (Merck, US). Five colonies larger than 1 mm in diameter were selected and placed in normal saline. The concentration of *C. albicans* solution was adjusted to 2 × 10^3^ CFU/mL.

### 2.2. Minimal inhibitory concentration (MIC)

The MIC was performed according to Clinical and Laboratory Standards Institute guidelines and previous report [22]. Sterile ultrapure water was used to dilute CGA-N12. Different concentrations of CGA-N12 with 6.0 mg/mL, 4.0 mg/mL, 2.0 mg/mL, 1.0 mg/mL, 0.4 mg/mL, 0.2 mg/mL, and 0.02 mg/mL were obtained. 100 µL prepared *C. albicans* solution and 100 µL different concentrations of CGA-N12 were added to a 96-well sterile plate. The mixed solution was incubated at 37°C for 24 h. Then, the mixed solution was coated on the SDA plate and cultured at 37’ for 24 h. Then, colony forming numbers were counted. Sterile ultrapure water without CGA-N12 was used as negative control. The experiment was repeated at least 3 times.

### 2.3. Minimal fungicidal concentration (MFC)

The MIC of CGA-N12 was used in this study. The culture medium was inoculated on SDA plate, and the growth of fungi was observed at 37°C for 24–48 hours. The minimum drug concentration corresponding to the sterile growth point was the MFC value, and the experiment was repeated at least 3 times.

### 2.4. Time-kill curve

The prepared *C. albicans* and CGA-N12 were mixed, and the final concentration of CGA-N12 was 12 mg/mL. After different incubation time (0 h, 2 h, 4 h, 6 h, 8 h, 10 h, 12 h, 14 h, and 16 h) at 37’ between *C. albicans* and CGA-N12, 100 µL medium was coated on the SDA plate. After incubation for 24 h at 37’, the colonies were counted using Image J software. The experiment was repeated at least 3 times, and time-kill curve was made.

### 2.5. Animal experiments

The animal experiment including drug-dose selections and animal group size was performed based on previous publications [23,24], and the protocol was modified after our pre-experiment. New Zealand white rabbits (2–3 kg) purchased from Charles River (Beijing, China) were used in this study. The animal experiments were divided into four groups (10 rabbits in each group): group control (treatment with normal saline), group *C. albicans* (treatment with *C. albicans*), group *C. albicans*+CGA-N12 (treatment with *C. albicans*+CGA-N12), and group *C. albicans*+Itraconazole (treatment with *C. albicans*+Itraconazole). The Itraconazole was purchased from Xian Janssen Pharmaceutical Ltd. (Xi’an, China). The concentration of *C. albicans* was diluted to 1 × 10^6^ CFU/mL. The animals were injected with *C. albicans* (1 mL×10^6^ CFU/mL) through their auricular vein. On the 10th day of *C. albicans* infection, rabbits were randomly dissected. The blood, liver, kidney, lung, brain and spleen were collected and cultured. If *C. albicans* was identified, the animal model was established successfully.

On the 10th day of *C. albicans* infection, animals in the group control and group *C. albicans* were treated with normal saline (15 mg/kg) intraperitoneally once every 2 days. Animals in the group *C. albicans*+CGA-N12 were injected with CGA-N12 (15 mg/kg) intraperitoneally once every 2 days. Animals in the group *C. albicans*+Itraconazole were injected with Itraconazole (15 mg/kg) intraperitoneally once every 2 days. The course of treatment was of 3 weeks. Serum was collected from each group on the 5th, 10th, and 20th day after the first treatment. Animals were sacrificed on the 30th day after the first treatment and their tissues were collected for histological examination. The animals’ experiments were performed based on some previous study [23,25] and the protocol was revised by our lab.

After injection treatment through their auricular vein, the inoculation sites, hair, temperature, survival status, and body change were monitored. The following conditions were set as humane end points in this study: rapid temperature decrease (more than 6’), rapid weight loss (more than 20%), systemic depilation, incurable long-term diarrhea, persistent vision dullness, mental malaise, drowsiness, and continuous lying down. Barbital sodium (100 mg/kg) intravenous injection was used for animal euthanasia.

### 2.6. Detection of cytokines

The levels of IL-10 (#PI525), TNF-α (#PT516), IL-6 (#PI335), and TNF-γ (#PT903) in the serum were measured using related ELISA kits according to the instruction. The ELISA kits were purchased from Beyotime (Beijing, China).

### 2.7. *Detection of* C. albicans *load*

The blood, lungs, liver, and kidneys were homogenized. A 1:10 dilution of the homogenate was plated onto SDA and cultured for 2 days at 37’. Then, the colony forming numbers were counted.

### 2.8 Hematoxylin-eosin (HE) staining

The tissues were fixed with formalin (4%) for 48 h and then embedded using optimal cutting temperature compound (OCT, Sigma, US). The tissues were cut into 8 μm sections. Three slides in each group were used for HE staining. Zeiss AxioVision (Jena) was used to acquire images.

### 2.9. Western blotting

Tissues were lysed using lysis buffer (Beyotime, Beijing, China). Protein concentrations were measured with a BCA kit (#ab102536, Abcam, UK). 40 µg protein was separated using SDS-PAGE, and transferred to a PVDF membrane (Millipore). The proteins were blocked with TBST buffer (#ab64202, abcam, UK) for 2 h. After washing twice, proteins were incubated with primary antibodies at 4°C overnight, and then incubated with secondary antibodies for 2 h after washing. Proteins were detected with an enhanced chemiluminescence detection kit (Thermo Fisher Scientific, USA), and bands was analyzed with ImageJ software. The antibodies used are: Rabbit monoclonal to SOCS1 (ab280886, 1:1000, abcam, UK), Rabbit monoclonal to GAPDH (ab181602, 1:1000, abcam, UK), Goat Anti-Rabbit IgG (ab96899, 1:2000, abcam, UK). GAPDH was used as a housekeeping control gene in the experiments as described previously [26].

### 2.10. qRT-PCR

RNA was extracted using Trizol reagent (TaKaRa, China). cDNA was synthesized using SuperScript™ IV (#18091050, ThermoFisher) and measured using RT-PCR with ChamQ^TM^ SYBR® qPCR Master Mix (Vazyme). The protocol of qRT-PCR was set as follows: 95°C, 30 seconds, 40 cycles (95°C, 3 seconds, and 60°C, 30 seconds). The primers used are SOCS1 (F: AGCTCCTTCCCCTTCCAGATT, R: CCACATGGTTCCAGGCAAGTA); GAPDH (F: ATGGGGAAGGTGAAGGTCG, R: TCGGGGTCATTGATGGCAACAATA). 2^−ΔΔCT^ method was used to analyze data.

### 2.11. Germ tube formation

*C. albicans* was inoculated into RPMI1640 medium preheated at 37°C (pH = 7.4, 10% FBS, and the final cell concentration was 5 x 10^6^/ml). CGA-N12 (6 mg/mL) was added and incubated using constant temperature oscillation culture at 37°C and 180 g/min. After 0 h and 3 h, a small amount of culture with inoculation ring on the glass slide was performed. The number of germ tubes produced in 100 cells was observed under microscope and counted randomly. The formation rate of germ tubes = (number of germ tubes/number of *C. albicans*) x 100%. Itraconazole (0.1 mg/mL) was used as the drug control and sterile PBS was used as the negative control. The experiment was repeated for 3 times in each group.

### 2.12. Statistical analysis

Data were shown as mean ±SD and analyzed with SPSS software (19.0, IBM, USA). Student’s t-test was used to analyze data between two groups. One-way ANOVA was used to analyze the data of multiple groups, followed by Dunnett’s t-test. p < 0.05 was considered to be statistically significant.

## 3. Results

### 3.1. *The MIC and MFC of CGA-N12 on* C. albicans

**Figure 1.**
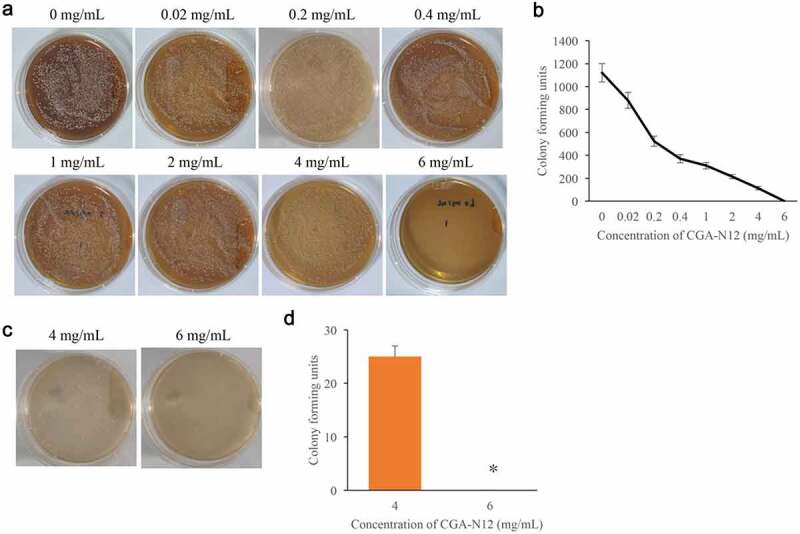
The MIC and MFC of CGA-N12 on *C. albicans* were measured. (a) MIC was measured through colony forming assays; (b) Colony-forming units were analyzed after treatment with different concentrations of CGA-N12; (c) MFC was measured through colony-forming assays; (d) Colony forming units were analyzed after treatment with different concentrations of CGA-N12. *P < 0.05 compared with group 4 mg/mL CGA-N12.

MIC and MFC are two common items used to evaluate the anti-fungal activity. Through colony forming assays, the MIC and MFC of CGA-N12 targeting *C. albicans* were detected. We found that with the increase of CGA-N12 concentration, the colony forming units decreased accordingly. The colony forming units became 0 when treated with 6 mg/mL CGA-N12 (Figure 1a-b), and the MIC of CGA-N12 was 6 mg/mL. Meanwhile, the MFC of CGA-N12 targeting *C. albicans* was proved to be 6 mg/mL (Figure 1c-d), and the chemical concentration of MIC and MFC is 0.45 mmol/L.

### 3.2. Germ tube formation rate and time-kill curves were measured

**Figure 2.**
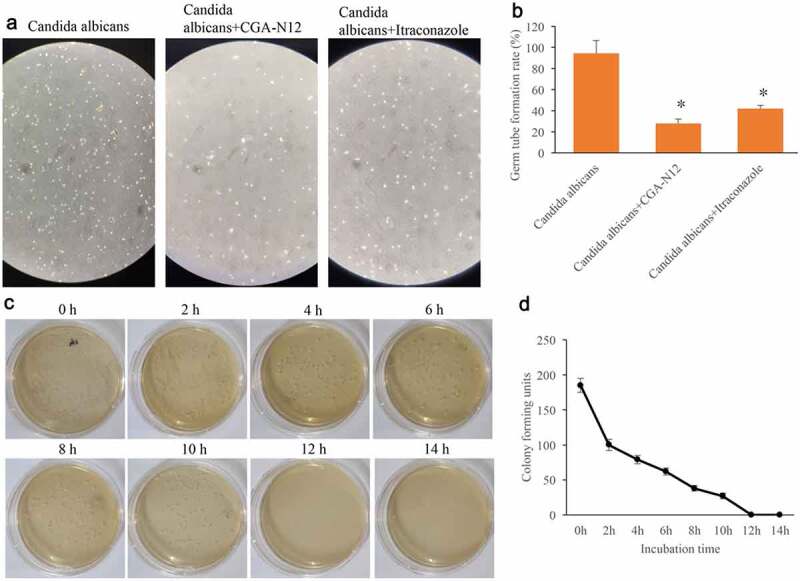
Germ tube formation rate and time-kill curves were measured. (a) Germ tube formation was measured after treatment with *C. albicans*, CGA-N12, and Itraconazole; (b) Germ tube formation rate was analyzed; (c) Time-kill curves were performed through colony forming assays; (d) Time-kill curves were analyzed. *P < 0.05 compared with group *C. albicans.*

Germ tube formation rate could reflect the growth speed of *C. albicans*. After treatment with CGA-N12 or Itraconazole, the germ tube formation rate of *C. albicans* was remarkably suppressed (Figure 2a-b), but no significant differences were observed between group CGA-N12 and Itraconazole. The data of time-kill curves indicated that CGA-N12 could significantly inhibit the growth of *C. albicans* (Figure 2c-d).

### 3.3. *Detection of* C. albicans *load in the tissues and inflammatory factors in the serum*

**Figure 3.**
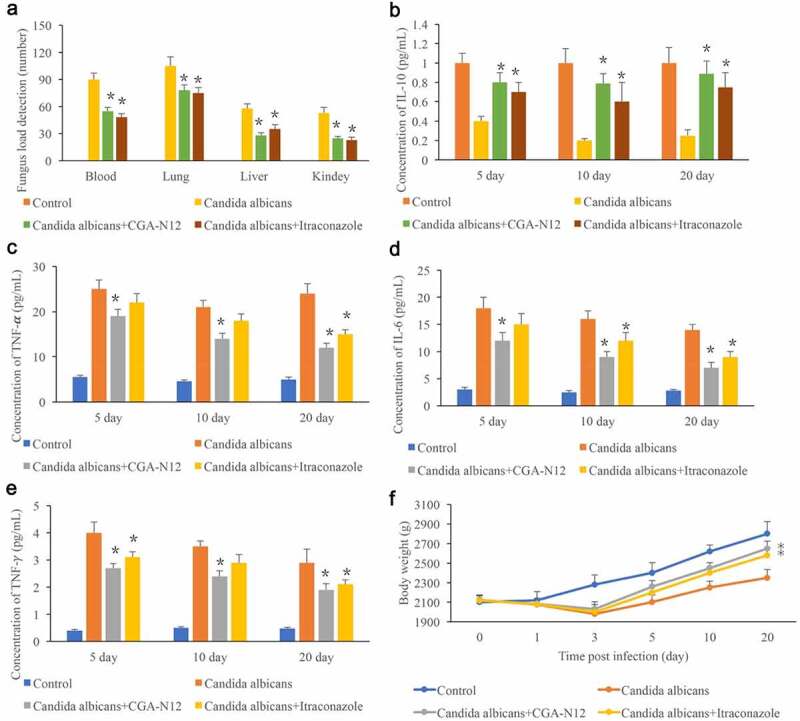
Detection of *C. albicans* load in the tissues and inflammatory factors in the serum. (a) Detection of *C. albicans* load in blood, lung, liver, and kidney; (b) The level of IL-10 in the serum was measured; (c) The level of TNF-α in the serum was measured; (d): The level of IL-6 in the serum was measured; (e): The level of TNF-γ in the serum was measured; (f): Body weight was recorded after infection. *P < 0.05 compared with group *C. albicans.*

The *C. albicans* load in blood, lung, liver, and kidney reflects the infection degree in vivo, and the *C. albicans* load in different tissues were measured. No *C. albicans* was detected in the group control, and remarkable increase of *C. albicans* was observed after the animals were administrated with *C. albicans*. Significant increase of *C. albicans* indicated that the *C. albicans* infected animal model was successfully established (Figure 3a). However, after treatment with CGA-N12 or Itraconazole, the levels of *C. albicans* in blood, lung, liver, and kidney were remarkably decreased (Figure 3a). In addition, the levels of TNF-α, IL-6, and TNF-γ in the serum were markedly increased in the group *C. albicans*, and the content of IL-10 was inhibited in the group *C. albicans*. However, both CGA-N12 and Itraconazole could effectively suppress the level of TNF-α, IL-6, and TNF-γ (Figure 3b-e). Therefore, CGA-N12 could inhibit the inflammation reaction caused by *C. albicans* in vivo. The body weight of rabbits was also recorded after infection. The weight of animals treated with CGA-N12 and Itraconazole increased significantly compared with group *C. albicans*.

### 3.4. *CGA-N12 significantly relieved histological damage caused by* C. albicans

**Figure 4.**
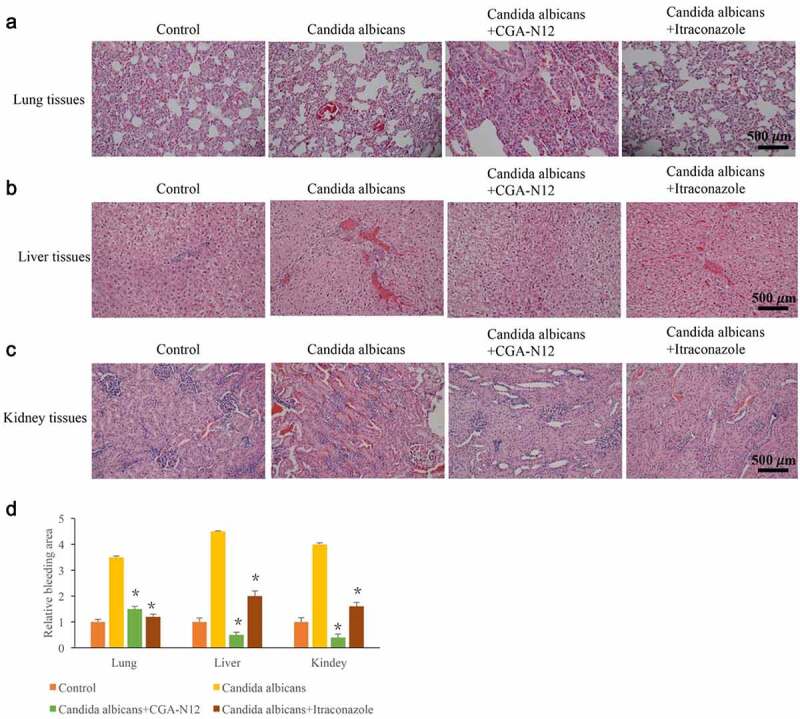
CGA-N12 significantly relieved histological damage caused by *C. albicans*. (a) Histological damage of lung tissues was remarkably relieved by CGA-N12; (b) Histological damage of liver tissues was remarkably relieved by CGA-N12; (c) Histological damage of kidney tissues was remarkably relieved by CGA-N12; (d) The bleeding areas were significantly decreased by CGA-N12.

Histological changes could directly reflect the influence of CGA-N12 on *C. albicans* infection. Disseminated *C. albicans* infection model in rabbit is used to study the effect of CGA-N12 on histological changes. After treatment with *C. albicans*, large area of inflammatory infiltration appeared in lung tissue, and a lot of bleeding points or bleeding blocks were observed. However, these pathological changes were significantly relieved by CGA-N12 and Itraconazole (Figure 4a). In the group *C. albicans*, the hepatocytes swelled like spheres and the cytoplasm became loose (Figure 4b). Meanwhile, a large area of inflammatory infiltration appeared in the kidney tissue, and diffuse bleeding was observed (Figure 4c). After treatment with CGA-N12, the inflammatory cells and bleeding points (Figure 4d) were significantly reduced.

### 3.5. CGA-N12 markedly increased the expression of SOCS1

**Figure 5.**
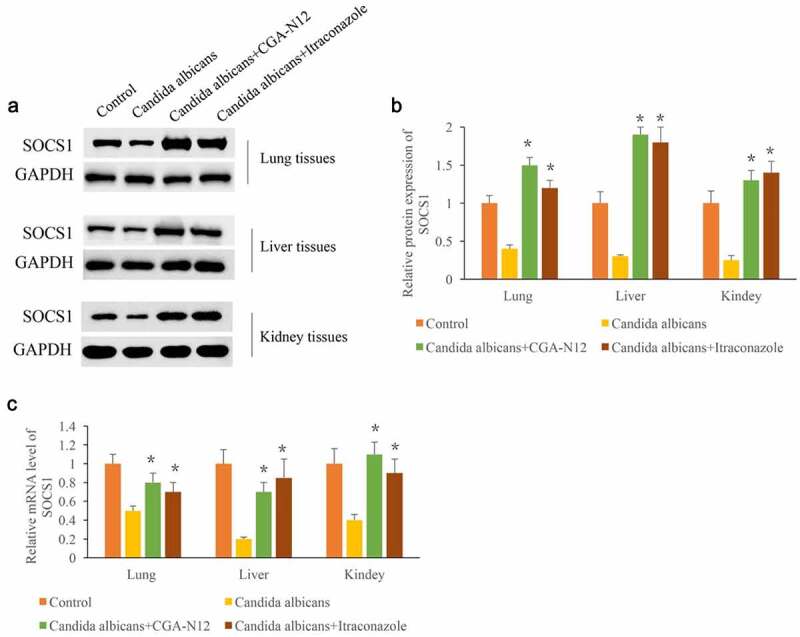
CGA-N12 markedly increased the expression of SOCS1. (a) The protein levels of SOCS1 in lung, liver, and kidney tissues were measured through Western blotting; (b) The protein levels of SOCS1 in lung, liver, and kidney tissues were analyzed; (c) The mRNA levels of SOCS1 in lung, liver, and kidney tissues were measured through qRT-PCR. *P < 0.05 compared with group *C. albicans.*

SOCS1 has proven to be closely linked with the inflammation response. After the administration of *C. albicans*, the protein and mRNA levels of SOCS1 in lung, liver, and kidney tissues were remarkably suppressed compared with group control (Figure 5a-c). However, the treatment with CGA-N12 or Itraconazole remarkably promoted both protein and mRNA expression of SOCS1 in lung, liver, and kidney tissues. Therefore, CGA-N12 might relieve the injury caused by *C. albicans*.

### 3.6. *Inhibition of* C. albicans *by CGA-N12 might be achieved through miR155/SOCS1*

**Figure 6.**
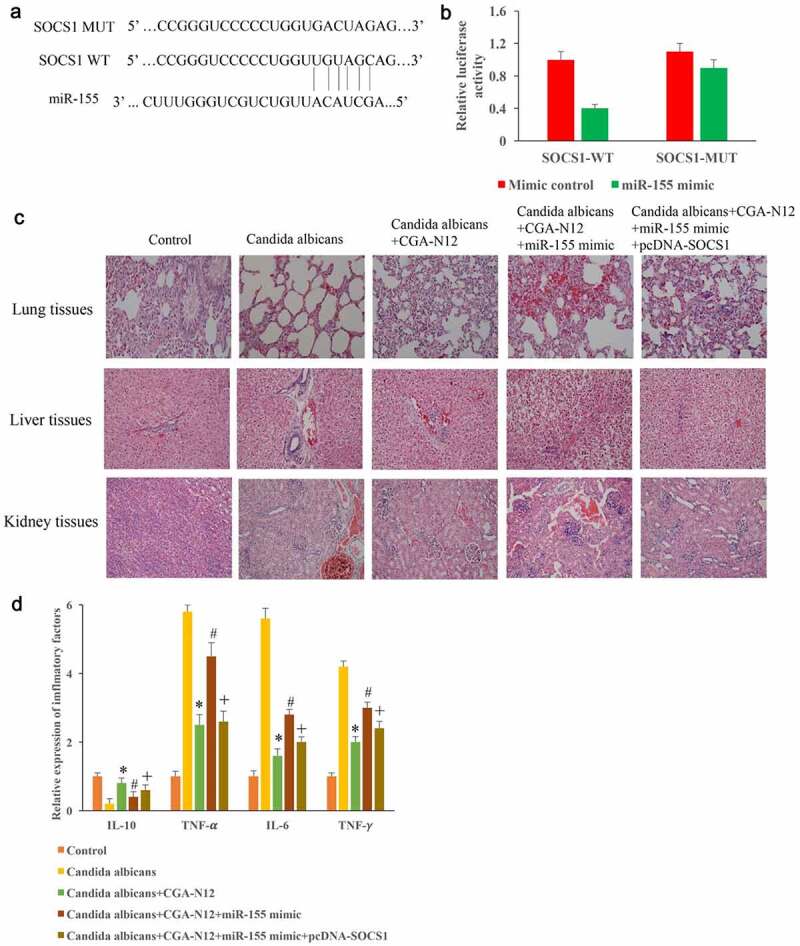
Inhibition of *C. albicans* by CGA-N12 might be achieved through miR155/SOCS1. (a) The binding site between SOCS1 and miR-155 was predicted; (b) The binding site between SOCS1 and miR-155 was identified; (c) Improvement of histological damage by CGA-N12 through miR155/SOCS1 axis; (f) Decreased of inflammatory factors by CGA-N12 was influenced by miR155/SOCS1 axis. *P < 0.05 compared with group *C. albicans*. # <0.05 compared with group *C. albicans*+CGA-N12. +<0.05 compared with group *C. albicans*+CGA-N12+ miR-155 mimic.

The binding site between SOCS1 and miR-155 was predicted and identified (Figure 6a-b). In addition, pcDNA-SOCS1 and miR-155 mimics were constructed and used to treat animals model induced by C. albicans. We found that the improvement of histological damages by CGA-N12 could be markedly reversed by overexpressing miR-155 in the (Figure 6c). However, transfection with pcDNA-SOCS1 markedly reversed the influence of miR-155 mimic (Figure 6c). Meanwhile, the content of IL-10 was decreased, the levels of TNF-α, IL-6, and TNF-γ in the serum were markedly increased in the group C. albicans, but they were significantly reversed after CGA-N12 treatment (Figure 6d). However, the influence of inflammatory factors by CGA-N12 were markedly changed by miR-155 mimic. In addition, the overexpression of SOCS1 significantly reversed the effect of miR-155 mimic, and suppressed the levels of inflammatory factors (Figure 6d).

### 3.7. *Influence of CGA-N12 on the germ tube formation rate of* Candida parapsilosis (C. parapsilosis) *were measured*

**Figure 7.**
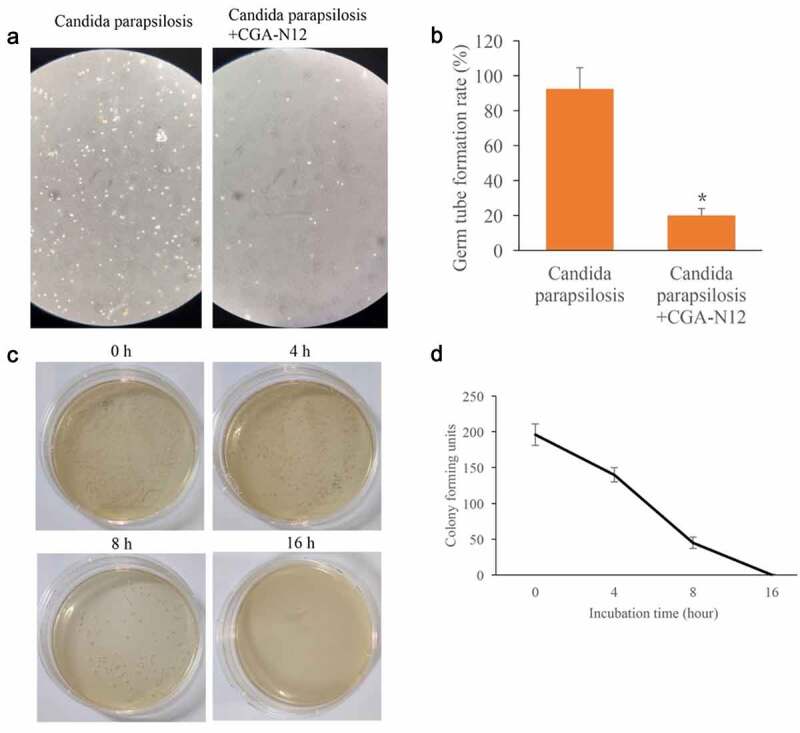
Germ tube formation rate and time-kill curves were measured. (a) Germ tube formation was measured after treatment with *C. parapsilosis*, and CGA-N12; (b) Germ tube formation rate was analyzed; (c) Time-kill curves were performed through colony forming assays; (d) Time-kill curves were analyzed. *P < 0.05 compared with group *C. parapsilosis.*

After treatment with CGA-N12, the germ tube formation rate of *C. parapsilosis* was markedly inhibited (Figure 7a-b). The data of time-kill curves indicated that CGA-N12 could completely inhibit the growth of *C. parapsilosis* after 16 h incubation (Figure 7c-d). These findings indicate that the anti-fungal effect of CGA-N12 is not specific only to *C. albicans*.

## 4. Discussion

AMPs have great potential for application due to the following advantages: good bactericidal effect, stability, less resistance to bacteria, less damage to cells, promotion of angiogenesis, wound healing and inflammation recovery. Therefore, AMPs are known as natural super antibiotics because of their incomparable advantages. According to the AMPs database (APD: http://aps.unmc.edu /AP/about.php), there are more than 2000 kinds of AMPs synthesized by natural or artificial methods, and nearly 1000 kinds of AMPs have antifungal activity. A variety of antimicrobial peptides have entered clinical trials. The phase IIIB clinical trials of rBP121, a derivative of porcine antimicrobial peptide IB-367, has been successfully completed [27]. PMX-30063/brilacidin used for skin infection treatment has passed the phase II b clinical trial [28]. Daptomycin, polymyxin and vancomycin have been used in the clinic [29,30].

As the first defense system of the body, AMPs do not only kill fungi directly but also eliminate pathogens by regulating the immune function of the body. AMPs have the ability of chemotaxis, and they can promote monocytes, mast cells, T-helper cells and neutrophils to gather to the inflammatory site, and stimulate the release of chemokines from monocytes and neutrophils of the host. Through these ways, AMPs play an important role in regulating inflammatory reaction [31]. AMPs can also inhibit the excessive activation of cytokines (proinflammatory cytokines, lipopolysaccharide, and other bacterial products) and reduce the damage to the host. AMPs have a unique antifungal mechanism, and they can play additive or synergistic effects when combined with other antibiotics. In addition, AMPs can interact with the matrix composed of polysaccharides and decompose biofilms.

CGA-N12 has proven to inhibit pathogenic microbes. For example, CGA-N12 could suppress *Candida tropicalis* by promoting the permeability of the mitochondrial membrane and further lead to mitochondrial potential dissipation [18,19]. In addition, CGA-N12 could suppress the cell wall synthesis of *Candida tropicalis* via interacting with KRE9 [32]. Meanwhile, CGA-N12 could increase metacaspase activation, Cyt c release, Ca^2+^ uptake in the cytoplasm and mitochondria, DNA fragmentation, and nuclear condensation [21]. These effects might account for the antifungal function of CGA-N12. Previous studies have indicated the effects of AMPs on *Candida tropicalis* infected rabbit. For example, CG(3)R(6)TAT nanoparticles self-assembled from a novel antimicrobial peptide presented effective treatment for *C. albicans* meningitis in rabbits [33]. We firstly demonstrated the role of CGA-N12 in *C. albicans* infected rabbits.

Suppressor of cytokine signaling (SOCS) is a family of immunosuppressive molecules, which can inhibit cytokine signal transduction. SOCS can regulate a variety of cytokines and participate in some inflammatory diseases. The SOCS1 gene is divided in 2 exons. The exon 1 contains the 5’untranslated region. The exon 2 contains part of 5’untranslated region, the translation initiation ATG, the stop codon, and the 3’untranslated region [34]. The primary accession number of SOCS1 is O15524 [35]. SOCS1 is an important attenuating factor of IFN and TLR mediated responses in various immune cells. In the macrophages, DC cells and fibroblasts of SOCS1 knockdown mice, significant high levels of proinflammatory cytokines including TNF-α, IL-6, IL-12 and IFN – γ were detected. In addition, SOCS1 knockout mice developed severe inflammation 3 weeks after birth and eventually died of multiple organ dysfunction syndrome [36]. Therefore, SOCS1 has the ability of inhibiting signal transduction of excessive cytokines. In this study, the protein and mRNA levels of SOCS1 were remarkably suppressed by CGA-N12, which might be the potential mechanism of inhibition of the inflammatory reaction by CGA-N12.

There are several limitations in this study. Firstly, we only investigated the regulation of CGA-N12 on miR-155/SOCS1, but did not further explore the targeting miRNA pathway, which needs further investigation. Secondly, one animal group inoculated with only CGA-N12, and a second one inoculated with itraconazole should be supplemented to investigate the influence of CGA-N12 and itraconazole on the host.

## 5. Conclusion

In summary, the significant suppression of *C. albicans* by CGA-N12 was demonstrated in vivo and in vitro. Meanwhile, CGA-N12 remarkably inhibited the levels of inflammation factors and promoted the expression of SOCS1, which might be the potential mechanism as to how CGA-N12 inhibits *C. albicans* growth and survival. This research might provide novel therapeutic strategies for the prevention and treatment of *C. albicans* infection.

## Data Availability

Data supporting this study has been presented in the manuscript; the data required by editor, reviewer and reader could be provided by the corresponding author.
